# Mosquito surveillance in maritime entry ports in Miami-Dade County, Florida to increase preparedness and allow the early detection of invasive mosquito species

**DOI:** 10.1371/journal.pone.0267224

**Published:** 2022-04-15

**Authors:** André B. B. Wilke, Chalmers Vasquez, Augusto Carvajal, Maday Moreno, William D. Petrie, John C. Beier

**Affiliations:** 1 Department of Public Health Sciences, Miller School of Medicine, University of Miami, Miami, FL, United States of America; 2 Miami-Dade County Mosquito Control Division, Miami, FL, United States of America; Fundacao Oswaldo Cruz Instituto Rene Rachou, BRAZIL

## Abstract

Invasive mosquito vector species have been inadvertently transported to new areas by humans for decades. Strong evidence supports that monitoring maritime, terrestrial, and aerial points of entry is an essential part of the effort to curb the invasion and establishment of invasive vector mosquito species. Miami-Dade County, Florida is an important operational hub for the cruise ship industry and leisure boats that routinely visit nearby areas in the Caribbean, and freight cargo ships transporting goods from Miami-Dade to Caribbean countries and vice versa. To deal with the increasing public health concern, we hypothesized that mosquito surveillance in small- and medium-sized maritime ports of entry in Miami-Dade is crucial to allow the early detection of invasive mosquito species. Therefore, we have selected 12 small- and medium-sized maritime ports of entry in Miami-Dade County with an increased flow of people and commodities that were not covered by the current mosquito surveillance system. Collection sites were comprised of two distinct environments, four marinas with international traffic of leisure boats, and eight maintenance and commercial freight cargo ship ports. Mosquitoes were collected weekly at each of the 12 collection sites for 24 hours for 6 weeks in the Spring and then for 6 additional weeks in the Summer using BG-Sentinel traps. A total of 32,590 mosquitoes were collected, with *Culex quinquefasciatus* and *Aedes aegypti* being the most abundant species totaling 19,987 and 11,247 specimens collected, respectively. Our results show that important mosquito vector species were present in great numbers in all of the 12 maritime ports of entry surveyed during this study. The relative abundance of *Cx*. *quinquefasciatus* and *Ae*. *aegypti* was substantially higher in the commercial freight cargo ship ports than in the marinas. These results indicate that even though both areas are conducive for the proliferation of vector mosquitoes, the port area in the Miami River is especially suitable for the proliferation of vector mosquitoes. Therefore, this potentially allows the establishment of invasive mosquito species inadvertently brought in by cargo freights.

## Introduction

Invasive vector mosquito species are a growing major public health concern [1]. Current estimates indicate that 129 countries and territories have conducive environments able to support the proliferation of invasive mosquito vector species [2–4]. The levels of mosquito-borne disease transmission continue to grow due to increased levels of proliferation and dispersion of invasive mosquito vector species across spatiotemporal scales greatly driving their presence and abundance in new areas [5–7].

Invasive mosquito vector species are being passively transported to new areas by humans for decades [8]. However, current levels of mobility with a worldwide increased flow of people and commodities have raised the risk of the introduction of invasive mosquito species considerably [9]. Several countries, such as New Zealand, The Netherlands, and Portugal, have been able to early detect and prevent the invasion of mosquito species that were being passively transported in tires, machinery, and by cars and commercial airplanes [10–12].

Unfortunately, that was not the case in many other countries where invasive mosquito vector species, such as *Aedes albopictus*, were able to successfully invade and become established [7]. *Aedes albopictus* is a primary vector of chikungunya, dengue, yellow fever, and Zika viruses [13], and subsequently to its invasion and establishment in most of Europe, arbovirus outbreaks were reported in Croatia, France, and Italy [14–16]. Strong evidence supports that monitoring maritime, terrestrial, and aerial points of entry is an essential part of the effort to curb the invasion and establishment of invasive vector mosquito species [17]. Different invasive mosquito species use different mechanisms to invade new areas and reliable and effective mosquito surveillance systems that can generate consistent and actionable data are paramount to avoid the spreading of invasive mosquito species into new areas [10, 18, 19].

Miami-Dade County, Florida is one of the most important gateways into the United States. Miami-Dade is located at a strategic geographic location being an important touristic and commercial entry point. Miami-Dade is one of the most important touristic destinations in the United States, receiving an average of over 120 million visitors every year [20]. Importantly, Miami-Dade has an increased flow of people and goods coming and going from the Caribbean region and Latin America. Miami-Dade is an important operational hub for the cruise ship industry, serving as the main port not only for cruise ships supplying the Caribbean and Gulf of Mexico region but also for leisure boats that routinely visit nearby areas in the Caribbean. Furthermore, freight cargo ships routinely transport goods from Miami-Dade to Caribbean countries and vice versa, including Haiti, Dominican Republic, and Cuba. Such high levels of human mobility and commodity trade increase the risk of the introduction of invasive species to Miami-Dade.

*Culex coronator* was able to successfully invade and colonize Miami-Dade County in less than 10 years from its first detection becoming one of the most abundant mosquito species with epidemiological relevance [21, 22]. Furthermore, the highly invasive species *Aedes vittatus* (Bigot, 1861), has been detected in Cuba and the Dominican Republic [23, 24]. In face of the high levels of human mobility and commodity trade between Miami-Dade and the Caribbean region, the introduction of *Ae*. *vittatus* to Miami-Dade is considered imminent.

Florida was the most affected state in the contiguous United States during the 2016 Zika virus outbreak totaling 256 locally transmitted cases [25, 26] with the Zika virus being introduced multiple times [27]. In 2020, 59 locally transmitted human cases of West Nile virus and 6 cases of dengue virus were reported in Miami-Dade by the Florida Department of Health and the CDC [28, 29]. To deal with the increasing public health concern, we hypothesized that mosquito surveillance in important small- and medium-sized maritime ports of entry in Miami-Dade is crucial to allow the early detection and elimination of invasive mosquito species. Therefore, our objective was to survey 12 small- and medium-sized maritime ports of entry in Miami-Dade County, Florida with an increased flow of people and commodities that were not covered by the current mosquito surveillance system [30].

## Methods

### Collection sites

**Fig 1:**
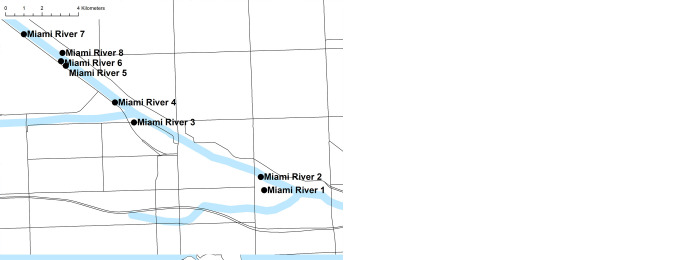
Map showing the location of the mosquito-surveyed maritime ports of entry in Miami-Dade, Florida. The figure was produced using ArcGIS 10.2 (Esri, Redlands, CA) using freely available layers from the Miami-Dade County’s Open Data Hub— https://gis-mdc.opendata.arcgis.com/.

Mosquitoes were collected in 12 small- and medium-sized maritime entry ports in Miami-Dade County, Florida. Collection sites were comprised of two distinct environments: (i) four marinas with international traffic of leisure boats–Marinas 1–4; and (ii) eight maintenance and commercial freight cargo ship ports–Miami River 1–8: **Marina 1:** Medium-sized marina owned and operated by the City of Miami. Adjacent to the Miami City Hall; **Marina 2:** Small-sized private marina with regular international boat traffic; **Marina 3:** Medium size marina with increased flow of tourists and leisure boats to the Caribbean region. Adjacent to the U.S. Coast Guard Sector Miami; **Marina 4:** Medium-sized private marina located in a touristic area with increased flow of international leisure boats; **Miami River 1:** Residential area adjacent to the Miami River with increased traffic of small and medium commercial and leisure boats; **Miami River 2:** Commercial medium-sized port specialized in the trade of used goods to Haiti; **Miami River 3:** Commercial medium-sized port specialized in the trade of goods to the Caribbean region; **Miami River 4:** Small-sized boat maintenance port adjacent to a car yard; **Miami River 5:** Medium-sized boat maintenance port adjacent to a car yard; **Miami River 6:** Medium-sized boat maintenance port adjacent to a scrap yard; **Miami River 7:** Medium-sized cargo port and storage; **Miami River 8:** Medium-sized scrap metal port (Fig 1).

### Mosquito collection

Mosquitoes were collected weekly at each of the 12 collection sites for 24 hours for 6 weeks in the Spring from April to May 2021 and then for 6 additional weeks in the Summer from June to July 2021 using BG-Sentinel traps (Biogents AG, Regensburg, Germany) baited with dry ice [31]. Mosquitoes were transported to the Miami-Dade County Mosquito Control Laboratory and subsequently morphologically identified to species using taxonomic keys [32]. “Since this study posed less than minimal risk to participants and did not involve endangered or protected species the Institutional Review Board at the University of Miami determined that the study was exempt from institutional review board assessment (IRB Protocol Number: 20161212).”

To compare the mosquito species composition between seasons (i.e., Spring and Summer) as well as marinas and commercial ports (i.e., Marinas 1–4 and Miami River 1–8) we performed a PERMANOVA with 9,999 permutations based on Bray-Curtis (abundance-based) and the Jaccard (incidence-based) indices [33–36]. Data was subsetted into two groups to compare variations in the mosquito species composition between Spring and Summer, and then we subsequently reorganized into 2 groups comprising marinas and commercial ports. Then we used the SIMPER method based on the Bray-Curtis index to assess which species has contributed the most to the observed differences between groups of samples [37]. Analyses were done using PAST v3.2 [38].

## Results

A total of 32,590 mosquitoes were collected, in the 12 maritime ports of entry surveyed during this project. *Culex quinquefasciatus* and *Aedes aegypti* were the most abundant species totaling 19,987 and 11,247 specimens collected, respectively. A total of 2,837 mosquitoes from 10 species were collected in the 4 marinas surveyed. *Culex quinquefasciatus* was the most abundant species with 1,925 specimens collected followed by *Ae*. *aegypti* with 633 specimens collected. Only one specimen of *Ae*. *albopictus* and one of *Cx*. *coronator* were collected in the Marinas. A total of 29,753 from 11 species were collected in the 8 ports in the Miami River. *Culex quinquefasciatus* was the most abundant species with 18,062 specimens collected followed by *Ae*. *aegypti* with 10,614. Differently from the Marinas, 122 *Ae*. *albopictus* and 16 *Cx*. *coronator* were collected in the Miami River. *Aedes bahamensis* and *Culex erraticus* were only found at the Marinas and *Anopheles atropos* was only collected at the Miami River (Table 1).

**Table 1:** Mosquitoes collected in 12 maritime ports of entry in Miami-Dade County, Florida.

Species		Marina 1	Marina 2	Marina 3	Marina 4	Miami River 1	Miami River 2	Miami River 3	Miami River 4	Miami River 5	Miami River 6	Miami River 7	Miami River 8
*Aedes aegypti*	F	40	103	4	247	1,049	1,155	530	1,081	990	605	356	333
	M	10	49	4	176	856	777	287	608	1,010	420	301	256
*Aedes albopictus*	F				1	1	5			54	16	8	13
	M						1		2	4		4	14
*Aedes bahamensis*	F							1	13	85	7	1	
	M									26	1	1	
*Aedes taeniorhyncus*	F		8		108		7			4			1
	M				5	1					1		1
*Aedes tortilis*	F				120	14			16	272	97	266	98
	M												
*Anopheles atropos*	F				1								
	M												
*Anopheles quadrimaculatus*	F				3							1	
	M												
*Culex coronator*	F		1					2		12	1		1
	M												
*Culex erraticus*	F									1			
	M												
*Culex nigripalpus*	F				3								8
	M												
*Culex quinquefasciatus*	F	741	217	65	229	959	1,418	881	1,664	3,321	2,402	664	1,165
	M	391	13	15	254	866	1,030	194	974	1,677	610	89	148
*Deinocerites cancer*	F	1	5		23	16							
	M												
Relative Abundance		1,183	396	88	1,170	3,762	4,393	1,895	4,358	7,456	4,160	1,691	2,038
Species Richness		3	5	2	9	6	4	4	5	8	7	6	7

F = females; M = males.

**Fig 2:**
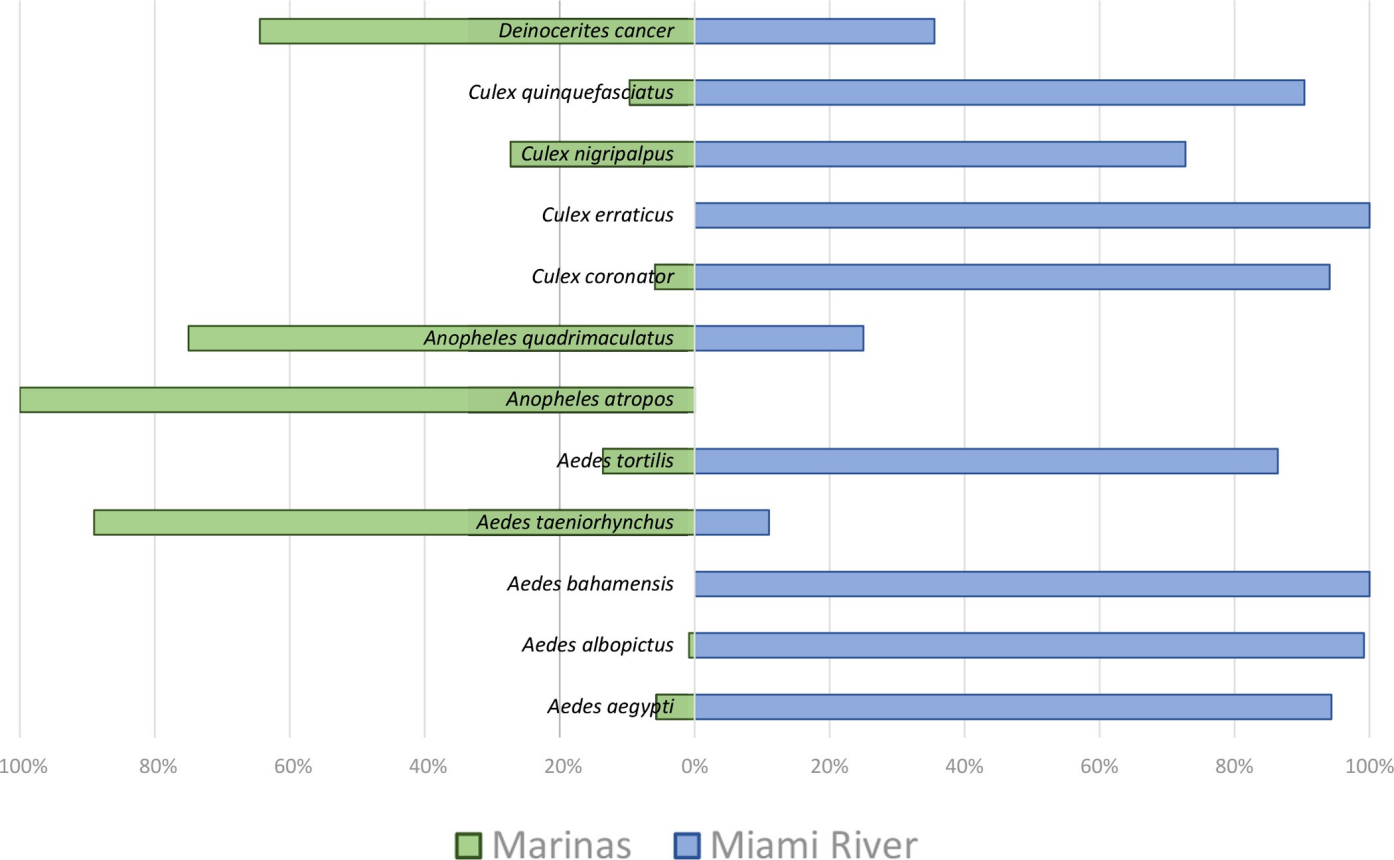
Relative proportion of mosquitoes collected in the two categories of maritime entry ports in Miami-Dade County, Florida, Marinas and commercial ports at the Miami River.

The relative proportion of mosquitoes collected in the Marinas and the Miami River varied greatly. On the one hand, *Aedes taeniorhynchus*, *Anopheles Atropos*, *Anopheles quadrimaculatus*, and *Deinocerites cancer* were more abundant at the Marinas. On the other hand, *Ae*. *aegypti*, *Ae*. *albopictus*, *Ae*. *bahamensis*, *Aedes tortillis*, *Cx*. *coronator*, *Cx*. *erraticus*, *Culex nigripalpus*, and *Cx*. *quinquefasciatus* were more abundant at the Miami River (Fig 2).

The PERMANOVA failed to yield significant results for the comparison between mosquito species composition collected in the Marinas and Miami River in different seasons, Spring and Summer based on both the Bray-Curtis (F = 1.225; *P* = 0.3008) and Jaccard (F = 0.9495; *P* = 0.432) indices. However, The PERMANOVA yielded significant results for the comparison between mosquito species composition collected in the Marinas and Miami River based on the Bray-Curtis (F = 6.767; *P* = 0.0037) and Jaccard (F = 3.373; *P* = 0.01) indices. The SIMPER analysis comparing the mosquito community composition of the Marinas and Miami River showed that *De*. *cancer*, *Ae*. *bahamensis*, *Ae*. *albopictus*, *Ae*. *tortilis*, and *Cx*. *coronator* contributed the most to the observed differences (Table 2).

**Table 2:** SIMPER (Similarity Percentage) analysis of which species contributed the most to the observed differences comparing maritime entry ports in Miami-Dade County, Florida.

Species	Average dissimilarity	Contribution %	Cumulative %
*Deinocerites cancer*	5.794	15.13	15.13
*Aedes bahamensis*	5.033	13.14	28.27
*Aedes albopictus*	4.872	12.72	40.99
*Aedes tortilis*	4.59	11.98	52.97
*Culex coronator*	4.108	10.73	63.7
*Aedes taeniorhynchus*	3.704	9.669	73.37
*Culex nigripalpus*	3.104	8.105	81.47
*Anopheles quadrimaculatus*	3.1	8.093	89.56
*Anopheles atropos*	2.803	7.318	96.88
*Culex erraticus*	1.194	3.118	100
*Culex quinquefasciatus*	6.98E-06	1.82E-05	100
*Aedes aegypti*	4.18E-06	1.09E-05	100

## Discussion

Mosquito surveillance in maritime ports of entry is crucial to allow the early detection of invasive mosquito species. Our results show that important mosquito vector species were present in great numbers in all of the 12 maritime ports of entry surveyed during this study, with *Cx*. *quinquefasciatus* and *Ae*. *aegypti* being the most abundant species. However, more importantly, the relative abundance of *Cx*. *quinquefasciatus* and *Ae*. *aegypti* was substantially higher in the Miami River than in the Marinas. These results are indicating that even though both areas are conducive for the proliferation of vector mosquitoes, the port area in the Miami River is especially suitable for the proliferation of vector mosquitoes. Therefore, potentially allowing the establishment of invasive mosquito species inadvertently brought in by cargo freights.

Our results also indicate that there were no significant differences in the mosquito community composition between the Marinas and the Miami River between spring and summer as seen previously as an overall trend for Miami-Dade County, in which *Cx*. *quinquefasciatus* usually peaks in abundance in March and April and *Ae*. *aegypti* in June and July [30]. On the other hand, despite the fact that *Ae*. *aegypti* and *Cx*. *quinquefasciatus* were found to be the dominant species in both the Marinas and the Miami River, the mosquito community composition was significantly different between these areas according to both the Bray-Curtis (abundance-based) and the Jaccard (incidence-based) indices.

The intense traffic of boats from Miami-Dade to the Caribbean region in the maritime ports of entry surveyed in this study alongside the elevated presence and abundance of vector species revealed a worrisome scenario. People coming and going from endemic areas may inadvertently become infected and introduce arboviruses to Miami-Dade to areas where workers spent a disproportionate amount of time outdoors and are exposed to large numbers of vector mosquito species. A similar scenario was documented during the Zika virus outbreak, in which construction workers were exposed to Zika-infected *Ae*. *aegypti* mosquitoes breeding in large numbers in a construction site [39, 40]. Furthermore, added to the risk of arbovirus introductions to Miami-Dade and the United States, there is also the risk of introduction of invasive mosquito species that are adapted to urban environments and are primary vectors of arboviruses (e.g., *Ae*. *vittatus*) that would further increase the risk of outbreaks in the region.

Our results indicate that all the surveyed ports of entry have (albeit at different levels) the potential to support high levels of mosquito vector populations and have the suitable conditions to allow a successful invasion and subsequent proliferation of invasive mosquito species that are adapted to urban environments. Invasive species could be brought to Miami-Dade either passively (i.e., transport of eggs or immature mosquitoes withing goods transported from the Caribbean region to Miami-Dade) or actively (i.e., adult mosquitoes seeking host actively board leisure boats and commercial freights and are inadvertently brought back to Miami-Dade). Therefore, mosquito surveillance should be intensified in small and medium maritime ports of entry to allow not only the early detection of invasive species but also to allow the development of effective control efforts to reduce the presence and abundance of dangerous mosquito vector species in these areas. Mosquitoes were not collected through all seasons, which may have led to the underestimation of species richness by not collecting rare species and failing to detect natural fluctuations in the mosquito community composition over time. However, our experimental design was appropriate to detect important mosquito vector species that should be considered by mosquito control strategies.

## Conclusion

Our results indicate that mosquito surveillance in small- and medium-sized maritime ports of entry are key not only to inform mosquito control operations to more effective control strategies to reduce populations of vector mosquito species in those areas and decrease the risk of arbovirus introductions but also to allow the early detection and elimination of invasive mosquito species inadvertently transported by leisure boats and cargo freight ships.

## References

[1] Schaffner F, Medlock JM, Van Bortel W. Public health significance of invasive mosquitoes in Europe. Clin Microbiol Infect. European Society of Clinical Microbiology and Infectious Diseases; 2013;19: 685–692. doi: 10.1111/1469-0691.12189 23574618

[2] Kraemer MUG, Sinka ME, Duda KA, Mylne A, Shearer FM, Brady OJ, et al. The global compendium of *Aedes aegypti* and *Ae*. *albopictus* occurrence. Sci Data. 2015;2: 150035. doi: 10.1038/sdata.2015.35 26175912 PMC4493829

[3] World Health Organization. Vector-borne diseases. 2020. Available at: https://www.who.int/news-room/fact-sheets/detail/dengue-and-severe-dengue

[4] Wilke ABB, Benelli G, Beier JC. Anthropogenic changes and associated impacts on vector-borne diseases. Trends Parasitol. 2021;37: 1027–1030. doi: 10.1016/j.pt.2021.09.013 34686421

[5] Wilke ABB, Beier JC, Benelli G. Complexity of the relationship between global warming and urbanization–an obscure future for predicting increases in vector-borne infectious diseases. Curr Opin Insect Sci. 2019;35: 1–9. doi: 10.1016/j.cois.2019.06.002 31279898

[6] Monaghan AJ, Eisen RJ, Eisen L, McAllister J, Savage HM, Mutebi JP, et al. Consensus and uncertainty in the geographic range of *Aedes aegypti* and *Aedes albopictus* in the contiguous United States: Multi-model assessment and synthesis. PLoS Comput Biol. 2019;15: 1–19. doi: 10.1371/journal.pcbi.1007369 31600194 PMC6786520

[7] Wilke ABB, Benelli G, Beier JC. Beyond frontiers: On invasive alien mosquito species in America and Europe. PLoS Negl Trop Dis. 2020;14: e0007864. doi: 10.1371/journal.pntd.0007864 31917804 PMC6952076

[8] Reiter P. *Aedes albopictus* and the world trade in used tires, 1988–1995: the shape of things to come? J Am Mosq Control Assoc. 1998;14: 83–94. 9599329

[9] Tatem AJ, Hay SI, Rogers DJ. Global traffic and disease vector dispersal. Proc Natl Acad Sci. 2006;103: 6242–6247. doi: 10.1073/pnas.0508391103 16606847 PMC1435368

[10] Ammar SE, Mclntyre M, Swan T, Kasper J, Derraik JGB, Baker MG, et al. Intercepted mosquitoes at New Zealand’s ports of entry, 2001 to 2018: Current status and future concerns. Trop Med Infect Dis. 2019;4: 101. doi: 10.3390/tropicalmed4030101 31284464 PMC6789606

[11] Ibañez-Justicia A, Gloria-Soria A, den Hartog W, Dik M, Jacobs F, Stroo A. The first detected airline introductions of yellow fever mosquitoes (*Aedes aegypti*) to Europe, at Schiphol International airport, the Netherlands. Parasit Vectors. 2017;10: 603. doi: 10.1186/s13071-017-2555-0 29221490 PMC5723084

[12] Osório HC, Zé-Zé L, Neto M, Silva S, Marques F, Silva AS, et al. Detection of the invasive mosquito species *Aedes* (*Stegomyia*) *albopictus* (diptera: Culicidae) in Portugal. Int J Environ Res Public Health. 2018;15: 1–9. doi: 10.3390/ijerph15040820 29690531 PMC5923862

[13] Benelli G, Wilke ABB, Beier JC. *Aedes albopictus* (Asian Tiger Mosquito). Trends Parasitol. 2020;36: 942–943. doi: 10.1016/j.pt.2020.01.001 32037135

[14] Poletti P, Messeri G, Ajelli M, Vallorani R, Rizzo C, Merler S. Transmission potential of chikungunya virus and control measures: The case of italy. PLoS One. 2011;6: e18860. doi: 10.1371/journal.pone.0018860 21559329 PMC3086881

[15] Gould EA, Gallian P, De Lamballerie X, Charrel RN. First cases of autochthonous dengue fever and chikungunya fever in France: From bad dream to reality! Clin Microbiol Infect. European Society of Clinical Infectious Diseases; 2010;16: 1702–1704. doi: 10.1111/j.1469-0691.2010.03386.x 21040155

[16] Gjenero-Margan I, Aleraj B, Krajcar D, Lesnikar V, Klobucar A, Pem-Novosel I, et al. Autochthonous dengue fever in Croatia, August-September 2010. Euro Surveill. 2011;16: 1–4. 21392489

[17] Schmidt TL, van Rooyen AR, Chung J, Endersby-Harshman NM, Griffin PC, Sly A, et al. Tracking genetic invasions: Genome-wide single nucleotide polymorphisms reveal the source of pyrethroid-resistant *Aedes aegypti* (yellow fever mosquito) incursions at international ports. Evol Appl. 2019;12: 1136–1146. doi: 10.1111/eva.12787 31297145 PMC6597869

[18] Müller P, Engeler L, Vavassori L, Suter T, Guidi V, Gschwind M, et al. Surveillance of invasive Aedes mosquitoes along swiss traffic axes reveals different dispersal modes for *Aedes albopictus* and *Ae*. *Japonicus*. PLoS Negl Trop Dis. 2020;14: 1–20. doi: 10.1371/journal.pntd.0008705 32986704 PMC7544034

[19] Fuehrer H-P, Schoener E, Weiler S, Barogh BS, Zittra C, Walder G. Monitoring of alien mosquitoes in Western Austria (Tyrol, Austria, 2018). PLoS Negl Trop Dis. 2020;14: e0008433. doi: 10.1371/journal.pntd.0008433 32574163 PMC7337398

[20] Bureau of Transportation Statistics. 2016 Annual and December U.S. Airline Traffic Data. 2017.Available at: https: //www.bts.gov/newsroom/2017-traffic-data-us-airlines-and-foreign-airlines-us-flights.

[21] Wilke ABB, Vasquez C, Cardenas G, Carvajal A, Medina J, Petrie WD, et al. Invasion, establishment, and spread of invasive mosquitoes from the *Culex coronator* complex in urban areas of Miami-Dade County, Florida. Sci Rep. 2021;11: 14620. doi: 10.1038/s41598-021-94202-8 34272411 PMC8285413

[22] Alto BW, Connelly CR, O’Meara GF, Hickman D, Karr N. Reproductive biology and susceptibility of Florida *Culex coronator* to Infection with West Nile Virus. Vector-Borne Zoonotic Dis. 2014;14: 606–614. doi: 10.1089/vbz.2013.1501 25072992 PMC4117262

[23] Pagac BB, Spring AR, Stawicki JR, Dinh TL, Lura T, Kavanaugh MD, et al. Incursion and establishment of the Old World arbovirus vector *Aedes* (*Fredwardsius*) *vittatus* (Bigot, 1861) in the Americas. Acta Trop. 2021;213: 105739. doi: 10.1016/j.actatropica.2020.105739 33159899

[24] Alarcón-Elbal PM, Rodríguez-Sosa MA, Newman BC, Sutton WB. The first record of *Aedes vittatus* (Diptera: Culicidae) in the Dominican Republic: Public health implications of a potential invasive mosquito species in the Americas. J Med Entomol. 2020;57: 2016–2021. doi: 10.1093/jme/tjaa128 32780102

[25] Likos A, Griffin I, Bingham AM, Stanek D, Fischer M, White S, et al. Local mosquito-borne transmission of Zika virus—Miami-Dade and Broward Counties, Florida, June–August 2016. Morbidity and Mortality Weekly Report. 2016;65: 1032–1038. doi: 10.15585/mmwr.mm6538e1 27684886

[26] Florida Department of Health. Available at: http://www.floridahealth.gov/diseases-and-conditions/mosquito-borne-diseases/_documents/week52arbovirusreport-12-31-16.pdf (2016).

[27] Grubaugh ND, Ladner JT, Kraemer MUG, Dudas G, Tan AL, Gangavarapu K, et al. Genomic epidemiology reveals multiple introductions of Zika virus into the United States. Nature. 2017;546: 401–405. doi: 10.1038/nature22400 28538723 PMC5536180

[28] Centers for Disease Control and Prevention. National arboviral surveillance system. 20202 Available at: https://wwwn.cdc.gov/arbonet/maps/ADB_Diseases_Map/index.html

[29] Florida Department of Health. Available at: http://www.floridahealth.gov/diseases-and-conditions/mosquito-borne-diseases/_documents/alert-dade-wnv-human-10-19-20.pdf (2020)

[30] Wilke ABB, Vasquez C, Medina J, Carvajal A, Petrie W, Beier JC. Community composition and year-round abundance of vector species of mosquitoes make Miami-Dade County, Florida a receptive gateway for arbovirus entry to the United States. Scientific Reports. 2019;9: 8732. doi: 10.1038/s41598-019-45337-2 31217547 PMC6584581

[31] Wilke ABB, Carvajal A, Medina J, Anderson M, Nieves VJ, Ramirez M, et al. Assessment of the effectiveness of BG-Sentinel traps baited with CO_2_ and BG-Lure for the surveillance of vector mosquitoes in Miami-Dade County, Florida. PLoS One. 2019;14: e0212688. doi: 10.1371/journal.pone.0212688 30794670 PMC6386269

[32] Darsie RF Jr., Morris CD. Keys to the Adult Females and Fourth Instar Larvae of the Mosquitoes of Florida (Diptera, Culicidae). 1st ed. Technical Bulletin of the Florida Mosquito Control Association; 2000.

[33] Anderson MJ. Permutational Multivariate Analysis of Variance (PERMANOVA). Statistics Reference Online. 2017; 1–15.

[34] Alencar J, De Mello CF, Guimarães AÉ, Gil-Santana HR, Dos Santos Silva J, Santos-Mallet JR, et al. Culicidae community composition and temporal dynamics in Guapiaçu ecological reserve, Cachoeiras de Macacu, Rio de Janeiro, Brazil. PLoS One. 2015;10: 1–16. doi: 10.1371/journal.pone.0122268 25815724 PMC4376767

[35] Ricotta C, Podani J. On some properties of the Bray-Curtis dissimilarity and their ecological meaning. Ecological Complexity. 2017;31: 201–205.

[36] Dávalos-Becerril E, Correa-Morales F, González-Acosta C, Santos-Luna R, Peralta-Rodríguez J, Pérez-Rentería C, et al. Urban and semi-urban mosquitoes of Mexico City: A risk for endemic mosquito-borne disease transmission. PLoS One. 2019;14: 1–19. doi: 10.1371/journal.pone.0212987 30840661 PMC6402764

[37] Clarke KR. Non-parametric multivariate analyses of changes in community structure. Austral Ecology. 1993;18: 117–143.

[38] Hammer Ø, Harper DATT, Ryan PD. PAST: Paleontological Statistics Software Package for Education and Data Analysis. Palaeontologia Electronica. 2001;4: 9.

[39] Mutebi J, Hughes HR, Burkhalter KL, Kothera L, Vasquez C, Kenney JL. Zika virus MB16-23 in mosquitoes, Miami-Dade County, Florida, USA, 2016. 2018;24: 4–6. 10.3201/eid2404.171919 PMC5875261 29400646

[40] Wilke ABB, Caban-Martinez AJ, Ajelli M, Vasquez C, Petrie W, Beier JC. Mosquito adaptation to the extreme habitats of urban construction sites. Trends in Parasitology. 2019;35: 607–614. doi: 10.1016/j.pt.2019.05.009 31230997

